# Grand Challenges for Nanoscience and Nanotechnology in Energy and Health

**DOI:** 10.3389/fchem.2017.00080

**Published:** 2017-10-31

**Authors:** Fan Zhang

**Affiliations:** Department of Chemistry, Fudan University, Shanghai, China

**Keywords:** nanotechnology, solar energy, nanomaterials, nanostructures, hydrogen energy, biomedicine, energy conversion, energy storage

During the past decade, new directions of modern research, broadly defined as nanoscale science and technology have emerged. Nanotechnology is not a separate scientific field, it is a complex platform for the existing disciplines of chemistry, physics, biology, medicine, neurology, information technology, and engineering, a new multidisciplinary scientific research area. In recent years, the nanotechnology has attract a great deal of attention in both synthesis methodologies and wide applications of medicine, energy, environmental, electronics etc. Despite of significant progress in nanotechnology and rise of many commercialized products involving nanomaterials, nanoscience, and technology are still facing many new challenges, especially in the areas of great concern to the public: energy and health.

## Nanomaterials for energy conversion

As versatile components of optoelectronic devices, the nanostructured materials attracted a great deal of attention due to its unique ability to manipulate light and control energy flow at nearly the atomic level (Ge and Yin, 2011; Linic et al., 2011). Actually, most of the so called next generation solar cells are based on the nanostructured materials, include those that are based on nanomaterials and/or nanostructures such as quantum dot solar cells, nanowire, mesoscopic nanostructures, and so on (Beard et al., 2012, 2014). They exhibit great promise of new conception or routes for converting solar energy into some other types of energy, such as electronic (Masuko et al., 2014; Mei et al., 2014), chemical fuels (Reece et al., 2011; Thomann et al., 2011), etc. However, there are still many challenges need to be addressed before they were fully used in the practical applications.

Most of today's commercial solar cells with typically efficiency of 10–25% are based the silicon (Green et al., 2015). To make solar energy economically competitive with other energy sources, it is critical find ways to lower the solar cell's costs by improve the efficiency. The nanotechnology provides us a valuable clue, because the nanomaterials can exhibit quite different and new properties compared with the corresponding bulk materials, which allow us to develop new ways to convert the solar energy into electricity or fuels (Nozik et al., 2010). Generally, there are three broad routes based on nanostructures are used to decrease the cost of solar cells (Shockley and Queisser, 1961): (1) decrease the usage of the materials, for example fabrication of the thin film solar cells; (2) increase the efficiency of the photovoltaic devices, for example fabrication of the multi-junction solar cells; (3) increase the stability and life cycle of the device. All the methods, individually or in combination, can lead greatly lower the costs of the solar energy.

For the front surface reflectivity in solar cell devices, nanostructured surfaces can effectively enhance the anti-reflection ability when the diameter of the nanostructure decreased below the wavelength of the incident light. If the active components can be patterned to the anti-reflection nanostructure, the usage of the solar light can be great enhanced, and the costly anti-reflection coatings and texturing can be avoided (Oh et al., 2012). In addition, because the patterned nanostructures can capture more light *via* light trapping so that less material is needed to absorb the solar flux (Garnett and Yang, 2010; Kelzenberg et al., 2010). New photovoltaic nanotechnologies should not only possess the potential to reduce the cost of module, but also to achieve power conversion efficiency beyond ~33% (Shockley–Queisser Efficiency Limit; Shockley and Queisser, 1961). Both the more efficient usage of high-energy photons and using the normally not converted photons with low energy (upconversion) can achieve higher limiting efficiencies. The two approaches to eliminate the energy losses are related the exploring of novel nanostructures (Beard et al., 2009, 2012; Choi et al., 2011; Wang et al., 2011; Krogstrup et al., 2013; Cirloganu et al., 2014). The device stability is another important factor need to be considered in the photovoltaic technologies for the decreasing of the cost of the solar energy (Zhang et al., 2013; Chuang et al., 2014; Ning et al., 2014).

The nanotechnology is a double-edged sword, some of the features that benefit to solar cells may also introduce additional challenges at the same time. The influence of surfaces and interfaces on recombination pathways are more important for nanocrystals due to its large surface/volume ratio. The migration of photo-excited charges can be hindered across nanocrystal grains, and the increased amount of surface states can deactivate the charge carriers. So, in a nanocrystal solid device, where each individual nanocrystal carries size dependent properties of the nanomaterials, the transport of charges is dominated by the interparticle medium. The surface ligands of the colloidal nanostructures should be well designed: (i) to make sure the stabilization of the colloidal stabilization, (ii) provide facile and stable charge transportation between the nanocrystals, and (iii) constructively supplement the properties of the nanocrystal solid.

While the new generation of photovoltaic cells (thin-film chalcogenides, hybrid perovskite materials, multi-junction solar cells, quantum dot-based solar cells, polymer solar cells etc.) exhibit great promise in the applications, it also presents challenges in the practical application. The challenges can be addressed in the following directions: (1) a simple fabrication processing with low manufacturing complexities and costs. Solution-based deposition and processing by using the nanoparticles inks is an appealing route. It has attracted a great deal of attention in the energy conversion, due to its unique properties including atmospheric pressure and low temperature processing, compatibility for the flexible substrates and large-area, high throughput, and so on. In addition, these approaches can be readily adapted for patterning materials without any subsequent processing steps. (2) One-dimensional nanomaterials can also offered new opportunities to design more efficient solar cells for enhancing solar cell efficiencies. By facilitating electron collection, transport and photon absorption, the one-dimensional nanostructures, including nanorods, nanotubes, nanowires etc. provide significant opportunities for improving solar cell efficiency. (3) The development of new materials with not only high photon absorption, electron transport, etc. but also heavy metal free is much more important for the practical application, because the many of the present energy conversion nanomaterial, such as perovskites, CdTe share a common disadvantage of environmentally hazardous heavy metal, which hinder the commercialization of them.

Beside the energy conversion, the energy economical consumption is also very important for the energy sustainable development. Among the straightforward successes of nanotechnology familiar to consumers, the light emitting devices (LED) with high electron to light conversion efficiency have been well developed in the past decade. The high luminescence efficiency and uniquely size-tunable color of solution processable semiconducting colloidal quantum dots (QDs) highlight the potential of QDLEDs for use in energy-efficient, solid state lighting and high color quality thin-film display. The key advantages of using quantum dots QDs in display and lighting applications, including their color purity, stability, and solution processability. From a device efficiency perspective, the three key challenges facing by QDLEDs are QDs photoluminescence quenching, poor photon outcoupling, and a limited understanding of the fundamental operating mechanisms of QDLEDs. Additionally, if the QDLED are to become a broad commercial reality, the operational lifetime and cost of QDLEDs must be addressed in the further research.

## Nanomaterials for energy storage

Besides the high efficient conversion of the solar energy, the storage of the converted energy is also critical desired, because the night or cloudy weather can interrupt solar energy's steadiness. We should capture and store the solar energy for the usage during the interruptions of the sun light. So, energy storage is very important for the efficient consumption of energy sources. As one of the most important constituent part, the nanomaterials are closely related to the energy conversion and storage. Owing to the innovation and advancement of materials science, the energy storage nanotechnologies have also been well-developed in the decades, especially the researches on hydrogen storage and Li-ion batteries.

Efficient hydrogen storage is regarded as the key challenge in large-scale applications of hydrogen energy. Hydrogen storage materials are the core technology for the storage of hydrogen sources with efficient and safe manner. To meet the stringent requirements of application of hydrogen energy, people has devoted many efforts to develop the potential materials for hydrogen storage (Liu et al., 2010). By setting up new reaction routes, several novel systems with well thermodynamic stability were developed based the hybrid nanomaterials. The intrinsic binding states can be tuned by substituting the cation/anion in host structures, which can induced the modification of the dehydrogenation thermodynamics and kinetics (Kang et al., 2008; Xiong et al., 2008; Hügle et al., 2009; Wang et al., 2009). For example, Wang et al. found that the peak dehydrogenation temperature can be effectively decreased by introducing potassium salts in the kinetic modification study of a Li-Mg-N-H material (Wang et al., 2009). By lowering the activation energy, catalytic activation also play a versatile and efficient role in the enhancement of the kinetically stability of the hydrogen sorption rate at the interface of solid, gas and liquid (Bluhm et al., 2006; Denney et al., 2006; Yao et al., 2007; Gunaydin et al., 2008; Yang et al., 2008; Berseth et al., 2009). Different from the heterogeneous catalysis based hydrogen storage materials, the more efficient homogeneous catalysis is characterized by molecule-level dispersion of catalyst within the host material, which can realized by directly dissolving ammonia borane into solvents. Denney et al.'s research result shows that the dehydrocoupling temperature of ammonia borane in THF, polyether etc. organic solutions can effectively decreased (typically 45–60°C) in the presence of transition-metal complexes (Denney et al., 2006). However, the separation processes in homogeneous catalysis are more complicated than that of the heterogeneous catalysis. To overcome the kinetic barrier in the mass transport and enhance the thermodynamic, the nanoscaling can provide an effective way (Wagemans et al., 2005; Li et al., 2007; Baldé et al., 2008). Balde et al. established the relationship between size and dehydrogenation performance in the carbon nanofibers supported NaAlH_4_ nanoparticles. With the decrease of particle size from 1–10 mm to 2–10 nm, the active energy can be successively reduced from 116 to 58 kJ mol^−1^ H_2_ (Baldé et al., 2008). With the assistant of the nano-confinement, the dilemma between the high capacity hydrogen storage and low dehydrogenation temperature may be addressed (Gutowska et al., 2005; Ingleson et al., 2009; Kim et al., 2009). Beside the chemisorption for the hydrogen storage, the physisorption possess the superiority of facile reversible storage at ambient temperature. The morphology and surface modification is crucial in the physisorption for achieving high hydrogen capacity (McKeown et al., 2006; Dinca and Long, 2007; Yang et al., 2007; Hamaed et al., 2008; Vitillo et al., 2008; Tsao et al., 2009). It has been demonstrated that a responsible pore diameter for the efficient hydrogen storage at liquid N_2_ temperature and ambient pressure is <1 nm. For example, the carbon materials (synthesized by using zeolite as the template) with surface area as high as 3,200 m^2^/g and narrow pore size distribution below 1 nm exhibit a high hydrogen binging energy of 8.2 kJ/mol (Yang et al., 2007). The last but not the least, the volumetric/weight hydrogen density, energy efficiency, thermodynamics, and kinetics of de-/rehydrogenation safety, cost etc. are other important factors need to overall considered for both chemisorption and physisorption in the developments of novel materials, nanostructures, and efficient synthetic techniques and strategies (Liu et al., 2010).

Li-ion batteries is one of the most important and widely used secondary batteries for the energy storage. The higher power/energy density, high speed recharge/discharge, and longer cycling life is much important for the newly emerging electronic devices, advanced communication and transportation facilities. In the past decades, many efforts were devoted to the development of the electrode materials with desirable electrochemical properties, including larger Li storage capacity, better cycling performance, and higher rate capability (Chen and Cheng, 2009). With the assistant of nanostructured materials with reduced Li-ion diffusion length and alleviated inner stress, the performance of the Li-ion batteries can be greatly improved in the rate capability and cyclability (Lee et al., 2003; Deng and Lee, 2008; Guo et al., 2008). It is very important for the development of the advanced Li-ion batteries to study the relationship between the performance and composition/nanostructure of an electrode material from the view of both theory and experiment. The compatibility of the electrode materials with electrolytes, redox sites, and surface conductivity can be greatly improved by surface engineering of the electrode materials, which further result in the improved electrochemical performance (Liu et al., 2010; Sun et al., 2016). On the other hand, rate capability and cycling performance can also be improved by growing the active nanomaterials directly on a current collector with enhanced electrical conductivity and bonding of the components (Zhang et al., 2006; Chan et al., 2008; Liu et al., 2010). Accompanied by the notable advantages, the shortcomings are also along with the subtly designed electrode nanomaterials, including large irreversible capacity, low packing density, complex synthesis processes, high cost, and so on, which further result in the limited practical applications until now. The major challenges at the material and electrode levels were large volume expansion and fracture, unstable SEI, slow electron/ion transport rate and movements of electrode atoms/molecules (Sun et al., 2016). Future works on understanding the fundamental electrode and materials chemistry taking place in these electrode systems are needed. Detailed information about the electrochemical mechanisms involved in these battery systems is still absent due to their complexity. Meanwhile, investigation of the ion and electron kinetic transport at the electrode/electrolyte interface is also important (Lin et al., 2014; Wang et al., 2014; Li et al., 2015).

## Nanomaterials for biomedicine

Theranostics is a term derived from therapy and imaging, which provide an integrated platform for the personalized medicine to meet the challenges in modern health care (Chen et al., 2011; Lammers et al., 2011). The theranostics is quite related to the biocompatible nanoparticle based nanomedicine, which contain both imaging and therapeutic nanocomponents. The radio-, gene-, or chemo therapeutics may be integrated in one nanoparticles. After combine it with the intrinsic optical, magnetic, etc. physicochemical properties or appropriate biomarkers, the nanocomposites would not only allow us to diagnose disease, but also evaluate treatment efficacy by track the nanoparticles' pharmacokinetics and the drugs releasing (Prabhu and Patravale, 2012; Li et al., 2014; Muthu et al., 2014).

The nano-theranostics will face a series of biological barriers during circulation in living subjects which will influence the nanoparticle delivery efficacy: the nanoparticles firstly should cross blood vessels, then escape the entrapment of organs and removal of phagocytic cells, finally reach the specific target (Kievit and Zhang, 2011; Blanco et al., 2015). An ideal theranostic nanoparticle should possess the following characters: rapid, selective, and high efficient accumulation in target diseased tissues; feedback the detailed information (biochemical, morphological, etc.) about the interest tissues or organs; release the guests (drugs, chemicals, etc.) with a controllable manner for the effective therapy; easy metabolism according to a safe with less side effects after its function completed.

It has been demonstrated that the circulation and metabolism in living subjects are profoundly effected by the Size (Tang et al., 2014), shape (Shah et al., 2011; Toy et al., 2014), rigidity (Ghassemi et al., 2012), charge (Fröhlich, 2012), and surface chemistry (Mout et al., 2012) of the nanoparticles (Albanese et al., 2012; Blanco et al., 2015). The theranostic nanoparticles are the complex of the delivery carriers and cargo, targeting ligands, and bio-imaging labels, which means that the clinical translation is nontrivial for these fancy materials (Ambrogio et al., 2011; Chow and Ho, 2013). There are many factors need to considered: the prerequisite robust scale-up synthesis; the biological responses for the theranostic nanoparticles including exposure levels, systemic accumulation, excretion profiles, tissue and organ distributions of test living subjects; the potential toxicity of the nanoparticle in short and long term (Prabhu and Patravale, 2012; Muthu et al., 2014).

## Conclusions

In the past decade, various nanostructures have been fabricated to address the significant material and applications challenges that exist in energy, environment, and health. Although, there are diverse specific requirements for nanomaterials in different applications, many commonality criterions for the research in the nanoscience can be built and summarized as following:

1. The structure-activity relationship between the nanomaterials and applications should be built firstly, which is critical for the design of the nanomaterials. A good performance in the application is our ultimate aim, so we should know what kind of materials are good for the improvement of the capability.
2. As the core components of nanotechnology, nanomaterials provides basic building blocks for fabricating complex devices with abundant functions. To better service for building up of the structure-activity relationship, the controllable targeting synthesis is another criterion in the nanoscience research.
3. Exploring synthetic routes to large-scale production with low cost is very important for the wide spread promotion of the new nanotechnology.
4. Last but not least, systemically evaluation on the toxicity and environmental risks of nanomaterials is essential.

## Author contributions

The author confirms being the sole contributor of this work and approved it for publication.

### Conflict of interest statement

The author declares that the research was conducted in the absence of any commercial or financial relationships that could be construed as a potential conflict of interest.
